# Effects of Chitooligosaccharide Coating Combined with Selected Ionic Polymers on the Stimulation of *Ornithogalum* *saundersiae* Growth

**DOI:** 10.3390/molecules22111903

**Published:** 2017-11-04

**Authors:** Piotr Salachna, Monika Grzeszczuk, Marcin Soból

**Affiliations:** 1 Department of Horticulture, West Pomeranian University of Technology, 3 Papieża Pawła VI Str., 71-434 Szczecin, Poland; monika.grzeszczuk@zut.edu.pl; 2 Center of Bioimmobilisation and Innovative Packaging Materials, West Pomeranian University of Technology, 35 Janickiego Str., 71-270 Szczecin, Poland; marcin.sobol@zut.edu.pl

**Keywords:** oligochitosan, gellan gum, plant promoter, physiological attributes, geophytes

## Abstract

Recently, agricultural and horticultural sectors have shown an increased interest in the use of biopolymers and their derivatives as growth biostimulators. So far, coating is a little known method of applying the biostimulators. Our three-year study investigated coating the bulbs of *Ornithogalum saundersiae* with chitooligosaccharide (COS), sodium alginate, carrageenan, gellan gum and xanthan gum. The coating method was based on the formation of polyelectrolyte complexes. The COS with 48,000 g mol^−1^ molecular weight was contained by means of controlled free-radical degradation. Biopolymer coatings stimulated plant growth and flowering, total chlorophyll content, total polyphenol content and the levels of nitrogen, phosphorus, potassium and boron. The plants grown from the bulbs coated with COS + gellan gum exhibited the most vigorous growth, were first to flower, showed the highest antioxidant activity (DPPH), and the greatest content of pigments, polyphenols, l-ascorbic acid, potassium, phosphorus, zinc and manganese. These results suggest COS formulated with gellan gum shows promise as a potential biostimulator of plant growth.

## 1. Introduction

Synthetic growth regulators used in agriculture and horticulture negatively affect the environment, hence the search for natural polysaccharides that can stimulate plant growth and development [1,2]. Chitosan, a derivative of chitin with β-1/4 GlcN and GlcNAc units, induces numerous biological responses in plants [3,4]. On an industrial scale, chitosan is obtained by chemical or enzymatic deacetylation of chitin, which occurs mainly in the shells of crustaceans [5]. It is used in plant production as a growth biostimulator [6] and elicitor of pathogen resistance [7,8]. The biological activity of chitosan depends on diverse factors as its morphological structure, degree of deacetylation and molecular weight [9,10]. Chitooligosaccharides (COS) are high molecular weight homo- or heterooligomers obtained from chitin or chitosan [11]. COS can stimulate plant growth [12,13], increase biomass accumulation [14], contents of photosynthetic pigments [15] and nutrients [16]. They may have stronger biological activity towards plants than chitosan and have huge practical application potential [17].

Other environmentally friendly biopolymers used in plant production include alginates and carrageenans [18]. Alginates are naturally occurring polysaccharide copolymers, consisting of residues of ß-d-mannuronic acid (M-blocks) and α-l-guluronic acid (G-blocks), joined together by glycosidic bonds. It is well established that sodium alginate (particularly in its depolymerized oligosaccharide form) may promote the growth of many plant species [19,20]. Carrageenans are linear polymers made up of galactose units. The stimulating effects of carrageenans and their derivatives on plant growth was demonstrated in such species as *Cicer arietinum*, *Zea mays* [21], *Mentha arvensis* [22], *Foeniculum vulgare* [23] and *Eucalyptus globulus* [24]. Another natural biopolymer is gellan gum, a water soluble anionic polysaccharide produced via aerobic fermentation by *Sphingomonas elodea*. Gellan gum used in micropropagation as an alternative for agar that can increase the regeneration potential of some plant species [25,26], which may indicate a biostimulating activity. Xanthan gum, a microbial desiccation-resistant polysaccharide, has been used with success as a gelling agent in micropropagated *Albizzia lebbeck*, *Datura innoxia* and *Calliandra tweedii* [27].

The biocompounds mentioned above may be used to form hydrogel coatings on the surface of plant organs in order to protect them from unfavorable external factors. This is a particularly promising patented method [28] based on polyelectrolyte complexes natural and chemically modified biopolymers [29,30]. The method involves two steps. At the first one, biological material is exposed to a low molecular weight ionic gel compound (e.g., an oligomeric cationic substance) and then, after drying, it is placed in an aqueous solution of a gelable polyelectrolyte (ionic biopolymer). Such coatings may accelerate plant growth [31] but since their effectiveness depends on their composition [32], more extensive research is necessary.

Demand for ornamental plants is steadily growing as a result of rising standard of living in many countries. The annual value of the ornamental bulb plants market alone is estimated to be one billion USD. Further development of this horticulture sector depends on introduction of new production technologies and supplementing the offer with new, attractive species [33]. Giant chincherinchee (*Ornithogalum saundersiae* Bak., Family Asparagaceae) is an example of a promising bulb plant cultivated mainly in Africa and Europe for cut flowers. In addition, *O. saundersiae* is a pot and garden plant [34] and it tolerates salinity [35]. Apart from its ornamental value *O. saundersiae* Bak. is an important raw material in pharmacological studies due to the presence of antineoplastic substances in its bulbs [36,37].

Our preliminary studies indicated that depolymerised chitosan enhanced the growth and flowering of *O. saundersiae*, regardless of the molecular weight [38]. In this work, we investigated the effects of four types of coatings containing COS, sodium alginate (A), carrageenan (C), gellan gum (G) and xanthan (X) on plant growth, physiological activity and the content of macro- and micronutrients of *O. saundersiae*.

## 2. Results and Discussion

### 2.1. Growth Attributes

COS and polysaccharides used for coating *O. saundesriae* bulbs stimulated most of the assessed morphological features of the plants (Table 1). 

Maximum values of growth related parameters were achieved in bulbs coated with COS + G. As compared with control, they had longer and more abundant leaves (by 28% and 36%, respectively), and produced more bulbs of greater fresh weight (by 138% and 74%, respectively). Moreover, the plants obtained from COS + G coated bulbs produced the best quality flowers with the longest inflorescence stems and the greatest number of flowers per inflorescence. Flowers of similarly high quality were produced by plants grown from bulbs coated with COS + C. Coating bulbs in biopolymers accelerated plant flowering by 8–12 days on average, depending on the type of coating. First to flower were the plants obtained from the bulbs coated with COS + G (Table 1).

### 2.2. Photosynthetic Pigment Contents

**Figure 1:**
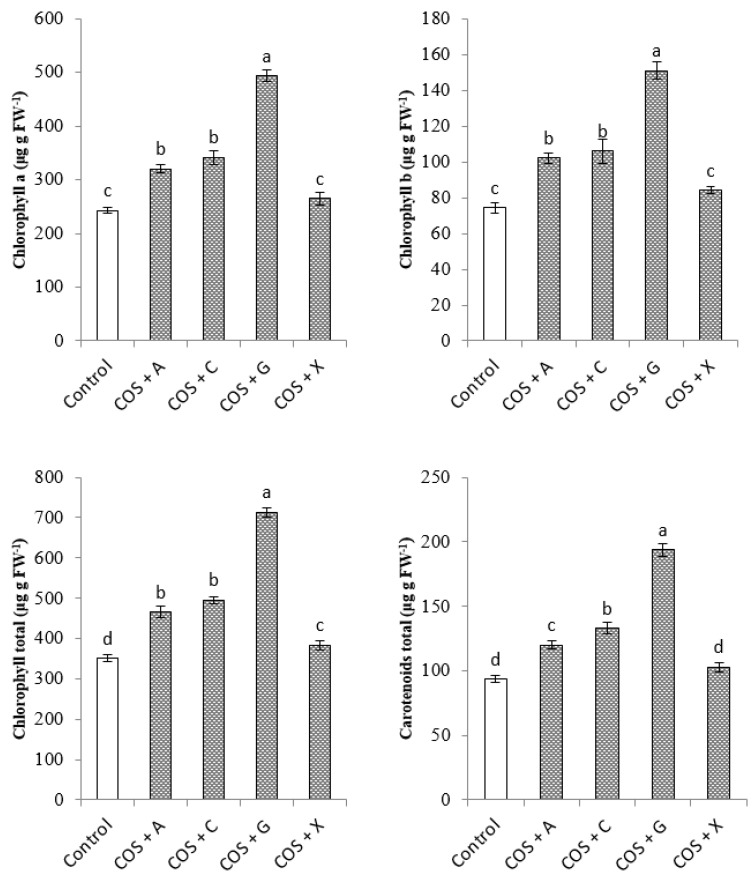
Effects of COS coating combined with selected ionic polymers on chlorophyll and carotenoids content of *Ornithogalum saundersiae*. Treatments: Non-coated bulbs (Control); COS + alginic acid sodium salt from brown alga (COS + A); COS + carrageenan iota (COS + C); COS + gellan gum (COS + G); COS + xanthan gum (COS + X). All data are means ± standard deviation (vertical bars). The columns with the different letters indicate significant differences (*p* ≤ 0.05).

Figure 1 shows the effects of COS and polysaccharides used for bulb coating on the content of photosynthetic pigments in the leaves of *O. saundersiae*. Plants obtained from the coated bulbs contained significantly more total chlorophyll, on average by 47% than the control ones. 

The highest content of total chlorophyll (713 μg g^−1^ FW), chlorophyll a (493 μg g^−1^ FW), chlorophyll b (151 μg g^−1^ FW) and carotenoids (194 μg g^−1^ FW) was determined for the plants treated with COS + G (Figure 1).

### 2.3. l-Ascorbic Acid, Total Polyphenols and Antioxidant Activity

**Figure 2:**
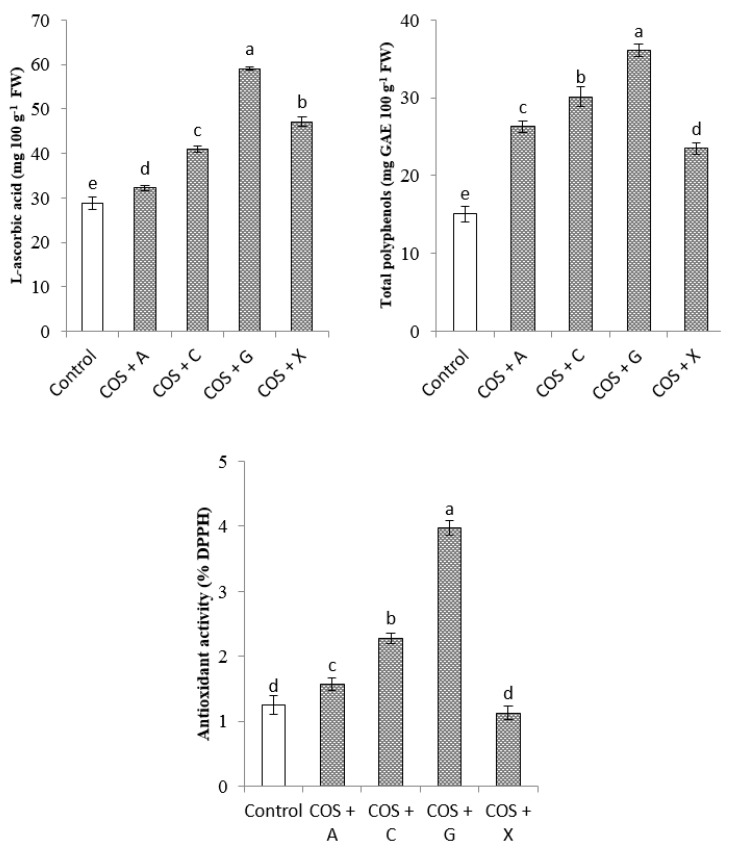
Effects of COS coating combined with selected ionic polymers on content of l-ascorbic acid, total polyphenols and antioxidant activity of *Ornithogalum saundersiae*. Treatments: Non-coated bulbs (Control); COS + alginic acid sodium salt from brown alga (COS + A); COS + carrageenan iota (COS + C); COS + gellan gum (COS + G), COS + xanthan gum (COS + X). All data are means ± standard deviation (vertical bars). The columns with the different letters indicate significant differences (*p* ≤ 0.05).

For the confirmation if bulb coating in polysaccharides induce changes in production of secondary metabolites in *O. saundersiae* leaf tissues, the content of total polyphenols as well as antioxidant activity (2,2-diphenyl-1-picrylhydrazyl (DPPH) free radical reduction), and the content of l-ascorbic acid were determined (Figure 2). The content of l–ascorbic acid was the highest in plants obtained from the bulbs coated with COS + G (59.1 mg 100 g^−1^ FW), and the lowest in control (28.8 mg 100 g^−1^ FW). 

The plants exposed to biopolymers contained more total polyphenols than the control ones. The highest content of total polyphenols (36.1 mg GAE 100 g^−1^ FW) and highest antioxidant activity (3.97% DPPH) was found in COS + G variant, whereas free DPPH radicals were the least effectively removed in control plants (1.29% DPPH).

### 2.4. Leaf Nutrient Concentrations

There were several changes in mineral accumulation in *O. saundersiae* leaves (Table 2 and Table 3). All plants grown from the coated bulbs contained more N, P and K in the leaves than controls. Those grown from the bulbs coated with COS + G were the richest in P and K. The plants grown from the control bulbs had the lowest levels of N, P and K (Table 2).

Accumulation of the micronutrients B, Zn and Mn in leaf tissue depended on bulb treatment before planting (Table 3). Plants obtained from the coated bulbs contained more B than the control ones. These increases were not significantly different between treatments. The highest levels of Zn and Mn in the leaves were found in the plants obtained from the bulbs coated with COS + G (Table 3).

### 2.5. Discussion

Chitosan, carrageenan, alginate and their depolymerized derivatives may affect plant growth and development, their morphology, as well as the course of life processes [18,39]. Our study demonstrated positive effects of all investigated coatings containing COS and different kinds of polysaccharides (sodium alginate, carrageenan, gellan gum and xanthan gum) on the leaf length of *O. saundesriae*, number of leaves, flowers per inflorescence and bulbs, length of scape, flowering, total chlorophyll content in the leaves, and the levels of N, P, K and B (Table 1, Table 2 and Table 3; Figure 1 and Figure 2). Similarly, enhanced growth, improved photosynthetic efficiency, chlorophyll content, and higher N, P, K, Ca and Mg levels were observed in the leaves of *Coffea canephora* var. Robusta sprayed with chitosan oligomers with molecular weight of about 2 kDa [16]. In hydroponically grown *Phaseolus vulgaris*, increasing doses of COS significantly modified morphological features of the plants and accumulation of P, K, Ca, Mg, Mo, B, Zn, Pb, Cd, Mn, Fe, Cu, Na and Al, without changing the content of chlorophyll a and b or carotenoids [11]. Wheat seedlings exposed to COS had, as compared with control, longer roots, higher photosynthesis efficiency and stomatal conductance index, contained more chlorophyll and proline and showed increased activity of SOD, CAT and POD [40]. In our experiments, the only coating type that did not show a significant effect on chlorophyll a, b, and carotenoids content, and antioxidant activity, was COS + X (Figure 1 and Figure 2). These differences could be attributed to the various physicochemical properties of polymers used for the coating preparation. Other reports have confirmed these results [32]. The enhancing effect of coating with different polymers on the vegetative growth and content of mineral compounds in the leaves of *O. saundersie* might be attributed to facilitation of nutrient uptake from soil mediated by COS. Besides, improved growth of plants may be due to positive effects of the biopolymers on the content of assimilation pigments. Increased content of mineral compounds and chlorophylls in the leaves certainly improves photosynthesis efficiency and nutrient supply.

Our results showed that coating bulbs in COS combined with selected ionic polymers stimulated an increase of the total polyphenols content in leaf tissues. Enhanced content of total polyphenols in plants obtained from the coated bulbs may indicate stronger antioxidant properties, including higher activity of antioxidant enzymes. Elevated secondary metabolism is often negatively correlated with plant growth [41] but in this study, the production of total polyphenols was stimulated by biopolymer coatings without a loss of biomass. Our results find support in the studies on *V. vinifera* cell culture [42], which confirm promoted growth and accumulation of phenolic acids, particularly 3-*O*-glucosyl-resveratrol, due to the chitosan and sodium alginate application. The mechanism of chitosan action in plants is still under investigation Apart from stimulating the enzymes degrading reactive oxygen species, the polymer is involved in intracellular signal transduction pathways of hydrogen peroxide and nitric oxide [6]. Recent studies in peas indicated that chitosan can interact with chromatin and alter gene expression [43].

Our study showed that of all tested biopolymer coatings, COS and gellan gum provided the strongest growth stimulation. These plants exhibited also the highest antioxidant activity and the greatest content of photosynthetic pigments in the leaves, total polyphenols, l-ascorbic acid, P, K, Zn and Mn. Considerable rise in antioxidant activity after exposition to COS and gellan gum was probably due to a stimulation of phenol biosynthesis. Results of previous investigations [44] indicated that foliar application of gellan gum enhanced vegetative growth and stomatal conductance in *Verbena bonariensis*. In in vitro plant cell and tissue culture techniques, gellan gum is used as a substitute to agar [26,27]. Gellan gum used as a gelling agent stimulated bud regeneration from pear leaves [45], promoted maturation of somatic embryos in *Pinus strobus* [46], improved the efficiency of micropropagation in *Arundo donax* [25], and increased shoot number in micropropagated *Eucomis autumnalis* subspecies *autumnalis* [26]. Contrary to that, a poor rotting response in three cultivars of spinano walut was observed when the plants were treated with Gelrite formulation containing gellan gum [47]. These reports assessed the effects of gellan gum on plant growth based on biometric measurements, whereas the mechanism of action of this polymer is yet to be elucidated. It can be assumed that the effect of gellan gum on plant growth and metabolism is due to the fact that the polymer contains water-soluble hormonal substances, macroelements, glycuronic acid, rhamnose and glucose [45,48]. In addition, gellan gum has a low content of phenols and high enzymatic activity, including superoxide dismutase (SOD) [49]. To better understand the effects of gellan gum on plant growth, further studies are recommended with a focus on physiological and biochemical processes triggered by this biopolymer. It should also be taken into account that the effectiveness of biopolymers as growth regulators depends to a large extent on plant genotype, form of the compound, its concentration or treatment of plant material.

## 3. Experimental

### 3.1. Preparation of Chitooligosaccharide

Chitosan with molecular weight of 333,000 g mol^−1^ and degree of deacetylation of 85% was used as a starting material (Yuhuan Ocean Biochemical, Taizhou, China). The COS with molecular weight of 48,000 g mol^−1^ was obtained via controlled radical degradation [30] with continuous addition of hydrogen peroxide at a final concentration of 6.2 mMol to 2.5% chitosan solution. The reaction was carried out at 80 °C for two hours. After that time, the reaction mixture was cooled, concentrated to approximately 15% (vacuum evaporator, RVO 200 A, INGOS, Prague, Czech Republic), precipitated in acetone, washed with pure acetone several times and dried at 50 °C for 4 h. Molecular weight of chitosan and COS samples was estimated by GPC S1000 HPLC pump, S2300 RI detector and a 20 µL loop, all from Knauer (Berlin, Germany). The column was a Tessek Separon HemaBio40 (Tessek, Prague, Czech Republic) and it was eluted at a flow rate of 1 mL/min with 0.33 M acetic buffer (pH 2.5) containing 0.1 M NaCl. Dextran standards (PSS, Mainz, Germany) were used for column calibration and as a relative reference for molecular weight calculation.

### 3.2. Plant Material and Experimental Design

The study was conducted in the years 2012–2014. Each year *O. saundersiae* bulbs with a perimeter of 14–16 cm were imported from The Netherlands by Ogrodnictwo Wiśniewski Jacek Junior Company (Warsaw, Poland). The bulbs were coated with biopolymers [31] prior to planting. The following experimental variants were tested:

1. control (non-coated bulbs)
2. COS + alginic acid sodium salt from brown alga (COS + A)
3. COS + carrageenan iota (COS + C)
4. COS + gellan gum (COS + G)
5. COS + xanthan gum (COS + X)

The bulbs were soaked for 10 min in 0.2% aqueous solution of COS. After drying (24 h), they were soaked for 30 s in 1% solutions of the remaining polysaccharides. Then, they were rinsed several times with water and dried for 24 h. Bulbs prepared this way had a 2 mm thick coating. Polyelectrolyte (Sigma Aldrich, Darmstad, Germany) solutions were prepared by solubilization with a magnetic stirrer. Each year a total of 40 bulbs were coated per variant, eight per each repetition.

### 3.3. Growing Conditions

On 10 May in all three years, coated bulbs were planted into polythene boxes 60 cm × 40 cm × 19 cm, filled with deacidified peat (Kronen, Cerkwica, Poland), pH 6.2, with the following mean content of macronutrients: 16 mg dm^−3^ N-NO_3_, 53 mg dm^−3^ P and 15 mg dm^−3^ K. The substrate was supplemented with a fertilizer Hydrocomplex (Yara International ASA, Oslo, Norway) at a dose of 5 g dm^−3^ that contained 12% N, 11% P_2_O_5_, 18% K_2_O, 2.7% MgO, 8% S, 0.015% B, 0.2% Fe, 0.02% Mn, and 0.02% Zn. Each box contained eight bulbs, planted at a spacing of 10 cm × 10 cm. The plants were cultivated under natural photoperiod under unheated plastic tunnel covered with a double layer of plastic film. Air temperature was controlled by vents that were opened when the temperature exceeded 20 °C. The plants were watered on average twice a week with a tap water in which mean ion concentration (mg dm^−3^) was as follows: 1.50 N-NO_3_, 1.3 P, 6.3 K, 94.7 Ca, 15.5, Mg, 22.0 Na, 22.0 Cl, 0.62 Cu, 0.41 Zn, 1.2 Fe, 196 HCO_3_, and 0.65 mS·cm^−1^ electrical conductivity.

### 3.4. Measurement of Growth Characteristics

The dates on which the first flower opened in each inflorescence were recorded on an ongoing basis. At this stage of plant development the number and length of leaves, the length of inflorescence stems (scape) and the number of flowers (florets) per inflorescence were determined. Each year the bulbs dug out in mid-October were counted and weighted.

### 3.5. Plant Analyses

Measurements were performed in well developed leaves from the central part of the plants. The leaves were collected during flowering from 20 randomly selected plants in each variant. All measurements were repeated four times.

#### 3.5.1. Pigments Assay

The content of total chlorophyll and chlorophyll a and b and carotenoids was determined according to Lichtenthaler and Wellburn [50]. Fresh plant material was extracted with 80% acetone. The samples were ground in a mortar in small amount of acetone and then aliquots were transferred to 50 cm^3^ volumetric flasks. Absorption of the extract was measured with Helios Gamma spectrophotometer (Thermo Spectronic, Cambridge, UK) at 441, 646, 652 and 663 nm. Pigment content was calculated using the following formulas:

chlorophyll a (µg/g FW) = (12.21 × E_663_ − 2.81 × E_646_) × (V/1000 × m),

chlorophyll b (µg/g FW) = (20.13 × E_646_ − 5.03 × E_663_) × (V/1000 × m),

total chlorophyll (µg/g FW) = (27.8 × E_652_) × (V/1000 × m),

total carotenoids (µg/g FW) = [(1000 × E_441_) − 3.27 × (12.21 × E_663_ − 2.81 × E_646_) − 104 × (20.13 × E_646_ − 5.03 × E_663_) ] × [V/1000 × (m × 229)],

where: E—extinction at specific wavelength, V—volume of a volumetric flask [cm^3^], and m—sample weight in g.

#### 3.5.2. Determination of l-Ascorbic Acid

The content of vitamin C as l-ascorbic acid was determined by Tillman’s method consisting in a reduction of a color solution of 2,6-dichlorophenolindophenol to a colorless leuco compound in the presence of l-ascorbic acid [51].

#### 3.5.3. Determination of Total Polyphenol

Total polyphenol content was determined spectrophotometrically with Folin-Ciocalteu reagent and gallic acid as standard, according to Singleton and Rossi [52]. A pulverized sample of plant material was extracted for 30 min with 70% methanol at the boiling point of the solvent. Aliquots of the cooled methanol extract were transferred into 100 cm^3^ volumetric flasks, the samples were filtered and the resulting extract was used for a quantitative determination of total polyphenol content. To this end, 5 cm^3^ of the extract was mixed with 75 cm^3^ of distilled water, 5 cm^3^ of Folin-Ciocalteu reagent, and 10 cm^3^ of saturated Na_2_CO_3_. The contents of the flask were supplemented with distilled water up to 100 cm^3^. The samples were kept for 60 min at room temperature, in darkness and then spectrophotometric (Helios Gamma, Thermo Spectronic) readings at 760 nm were performed.

#### 3.5.4. Determination of Antioxidant Activity

Antioxidant activity was determined by reduction of 2,2-diphenyl-1-picrylhydrazyl ( DPPH) free radicals, according to Yen and Chen [53], and DPPH inhibition percentage was calculated according to the formula provided by Rossi et al. [54]. At first, a reagent containing a solution of free radicals was prepared. To this end, 0.012 g of DPPH were weighed and quantitatively transferred to a 100 mL volumetric flask that was then filled up to the mark with methanol (100%). The solution was dissolved with a Sonic-3 ultrasonic washer. The examined sample (At) was made up in a test tube by combining 1 mL of the sample diluted (400 times) in methanol, 3 mL of methanol, and 1 mL of DPPH solution. The sample was stirred and after 10 min its absorbance was read with a spectrophotometer at 517 nm. The percentage of DPPH inhibition was calculated as per the following formula:

% of DPPH inhibition = 100 − [(At/Ar) × 100],

where: At—absorbance of the test solution and Ar—absorbance of the reference solution.

#### 3.5.5. Nutrient Analysis

Collected leaves were washed with water and dried at 80 °C for 48 h. Each year about 500 g of raw plant material were used in the analyses. After mineralization in a mixture of H_2_SO_4_ and H_2_O, the content of following nutrients was determined: total nitrogen (N) by Klejdahl’s method, potassium (K) by flame photometry AFP-100, phosphorus (P) and boron (B) by a Spectronic GENESYS 6 UV-Visible spectrophotometer (Thermo Electron Scientific Instruments Corporation, Cambridge, UK), magnesium (Mg), calcium (Ca), copper (Cu), zinc (Zn), manganese (Mn) and iron (Fe) by atomic absorption spectrophotometry ASS 30 [55]. All determinations were performed in four repetitions.

#### 3.5.6. Statistical Analysis

The experiment was a univariate one in a random block design. Average results of the three years of the study were verified statistically using the variance analysis model (ANOVA). The resulting means were grouped using Tukey’s test for the significance level α = 0.05. Statistical analysis was performed in Statistica 12.0 (StatSoft, Cracov, Poland).

## 4. Conclusions

The study demonstrated a stimulating effect of coatings composed of COS and selected ionic biopolymers on the growth of *O. saundersiae*. Bulb coating improved the morphological features of the plants, which bloomed earlier and contained more total chlorophyll, total polyphenols and N, P, K and B than control plants. The most favorable effects concerning growth, antioxidant activity, content of pigments, polyphenols, l-ascorbic acid, P, K, Zn and Mn were observed for COS + gellan gum coating. Apart from its cognitive aspects, the study has also a significant practical potential, as it is the first time that gellan gum was reported as plant growth biostimulator in bulbous plant production Raw materials used for coating bulbs are safe for humans, they do not pollute the environment, and help us to reduce the use of harmful chemicals in the agriculture.

## Figures and Tables

**Table 1:** Effects of COS coating combined with selected ionic polymers on plant growth of *Ornithogalum saundersiae*.

Parameters	Coating Type				
	Control	COS + A	COS + C	COS + G	COS + X
Leaf length (cm)	69.0 ± 2.0 ^d^	73.8 ± 1.25^c^	84.8 ± 0.65 ^b^	88.5 ± 0.50 ^a^	75.1 ± 1.55 ^c^
Number of leaves	6.25 ± 0.05 ^d^	7.83 ± 0.28 ^b^	8.00 ± 0.26 ^a,b^	8.50 ± 0.50 ^a^	7.00 ± 0.10 ^c^
Days to anthesis	80.2 ± 2.05 ^a^	74.3 ± 0.98 ^c^	72.0 ± 2.26 ^c^	68.0 ± 1.60 ^d^	77.2 ± 1.10 ^b^
Scape length (cm)	109 ± 5.51 ^d^	142 ± 2.25 ^b^	146 ± 4.04 ^a^	149 ± 5.50 ^a^	129 ± 5.51 ^c^
Number of florets	65.7 ± 1.52 ^c^	71.7 ± 1.52 ^b^	80.6 ± 5.85 ^a^	78.3 ± 1.53 ^a^	69.0 ± 1.00 ^b,c^
Bulbs fresh weight (g)	156 ± 6.13 ^d^	189 ± 8.14 ^c^	226 ± 9.51 ^b^	272 ± 7.54 ^a^	191 ± 5.20 ^c^
Number of bulbs	1.17 ± 0.31 ^c^	1.59 ± 0.42 ^b^	1.70 ± 0.45 ^b^	2.78 ± 0.68 ^a^	1.42 ± 0.11 ^b,c^

Treatments: Non-coated bulbs (Control); COS + alginic acid sodium salt from brown alga (COS + A); COS + carrageenan iota (COS + C); COS + gellan gum (COS + G), COS + xanthan gum (COS + X). All data are means ± standard deviation. Different letters in the same row indicate significant differences (*p* ≤ 0.05).

**Table 2:** Effects of COS coating combined with selected ionic polymers on macronutrients concentrations (g kg^−1^ DW) in the leaves of *Ornithogalum saundersiae*.

Nutrients	Coating Type				
	Control	COS + A	COS + C	COS + G	COS + X
N	21.1 ± 0.91 ^b^	24.0 ± 1.10 ^a^	24.5 ± 0.90 ^a^	25.1 ± 0.73 ^a^	23.9 ± 0.70 ^a^
P	3.40 ± 0.10 ^c^	5.01 ± 0.41 ^b^	5.14 ± 0.80 ^b^	5.60 ± 0.30 ^a^	4.93 ± 1.30 ^b^
K	30.9 ± 2.27 ^d^	42.9 ± 2.91 ^b,c^	45.4 ± 2.56 ^b^	49.9 ± 1.73 ^a^	40.7 ± 3.74 ^c^
Ca	12.7 ± 1.63 ^a^	13.9 ± 1.67 ^a^	12.3 ± 0.61 ^a^	15.3 ± 0.97 ^a^	13.4 ± 0.65 ^a^
Mg	2.70 ± 0.20 ^a^	2.70 ± 0.41 ^a^	2.61 ± 0.10 ^a^	2.53 ± 0.51 ^a^	2.80 ± 0.30 ^a^

Treatments: Non-coated bulbs (Control); COS + alginic acid sodium salt from brown alga (COS + A); COS + carrageenan iota (COS + C); COS + gellan gum (COS + G); COS + xanthan gum (COS + X). All data are means ± standard deviation. Different letters in the same row indicate significant differences (*p* ≤ 0.05).

**Table 3:** Effects of COS coating combined with selected ionic polymers on micronutrients concentrations (mg kg^−1^ DW) in the leaves of *Ornithogalum saundersiae*.

Nutrients	Coating Type				
	Control	COS + A	COS + C	COS + G	COS + X
B	20.9 ± 0.64 ^b^	28.7 ± 0.35 ^a^	29.6 ± 0.42 ^a^	28.9 ± 1.18 ^a^	31.8 ± 0.76 ^a^
Cu	2.32 ± 0.30 ^a^	2.45 ± 0.14 ^a^	2.38 ± 0.23 ^a^	2.35 ± 0.18 ^a^	2.49 ± 0.39 ^a^
Zn	30.0 ± 2.05 ^b^	33.3 ± 2.83 ^a,b^	29.4 ± 0.64 ^b^	39.3 ± 3.04 ^a^	30.0 ± 3.82 ^b^
Mn	14.3 ± 1.98 ^b^	16.5 ± 0.72 ^a,b^	15.8 ± 0.07 ^a,b^	18.1 ± 1.70 ^a^	14.8 ± 0.21 ^b^
Fe	56.7 ± 5.04 ^a^	47.8 ± 4.69 ^a^	49.7 ± 6.70 ^a^	51.9 ± 5.04 ^a^	53.1 ± 5.74 ^a^

Treatments: Non-coated bulbs (Control); COS + alginic acid sodium salt from brown alga (COS + A); COS + carrageenan iota (COS + C); COS + gellan gum (COS + G); COS + xanthan gum (COS + X). All data are means ± standard deviation. Different letters in the same row indicate significant differences (*p* ≤ 0.05).

## References

[1] Cabrera J.C., Wégria G., Onderwater R.C.A., González G., Nápoles M.C., Falcón-Rodríguez A.B., Costales D., Rogers H.J., Diosdado E., González S. (2013). Practical use of oligosaccharins in agriculture. Acta Hortic..

[2] Kashyap P.L., Xiang X., Heiden P. (2015). Chitosan nanoparticle based delivery systems for sustainable agriculture. Int. J. Biol. Macromol..

[3] Hadwiger L.A. (2013). Plant science review: Multiple effects of chitosan on plant systems: Solid science or hype. Plant Sci..

[4] Malerba M., Cerana R. (2016). Chitosan effects on plant systems. Int. J. Mol. Sci..

[5] Katiyar D., Hemantaranjan A., Singh B. (2015). Chitosan as a promising natural compound to enhance potential physiological responses in plant: A review. Indian J. Plant Physiol..

[6] Pichyangkura R., Chadchawan S. (2015). Biostimulant activity of chitosan in horticulture. Sci. Hortic..

[7] Pospieszny H., Chirkov S., Atabekov J. (1991). Induction of antiviral resistance in plants by chitosan. Plant Sci..

[8] Wiśniewska-Wrona M., Niekraszewicz A., Ciechańska D., Pospieszny H., Orlikowski L.B. (2007). Biological properties of chitosan degradation products. Pol. Chitin Soc. Monogr..

[9] Luan L.Q., Ha V.T.T., Nagasawa N., Kume T., Yoshii F., Nakanishi T.M. (2005). Biological effect of irradiated chitosan on plants in vitro. Biotechnol. Appl. Biochem..

[10] Salachna P., Zawadzińska A. (2014). Effect of chitosan on plant growth, flowering and corms yield of potted freesia. J. Ecol. Eng..

[11] Lodhi G., Kim Y.-S., Hwang J.-W., Kim S.-K., Jeon Y.-J., Je J.-Y., Ahn C.-B., Park P.-J. (2014). Chitooligosaccharide and its derivatives: Preparation and biological applications. Biomed. Res. Int..

[12] Nge K.L., Nwe N., Chandrkrachang S., Stevens W.F. (2006). Chitosan as a growth stimulator in orchid tissue culture. Plant Sci..

[13] Winkler A.J., Dominguez-Nuñez J.A., Aranaz I., Poza-Carrión C., Ramonell K., Somerville S., Berrocal-Lobo M. (2017). Short-chain chitin oligomers: Promoters of plant growth. Mar. Drugs.

[14] Wang Z., Zhao Y., Wei H. (2017). Chitosan oligosaccharide addition affects current-year shoot of post-transplant Buddhist pine (*Podocarpus macrophyllus*) seedlings under contrasting photoperiods. IFOREST.

[15] Zou P., Li K., Liu S., Xing R., Qin Y., Yu H., Zhou M., Li P. (2015). Effect of chitooligosaccharides with different degrees of acetylation on wheat seedlings under salt stress. Carbohydr. Polym..

[16] Dzung N.A., Khanh V.T.P., Dzung T.T. (2011). Research on impact of chitosan oligomers on biophysical characteristics, growth, development and drought resistance of coffee. Carbohydr. Polym..

[17] Zou P., Tian X., Dong B., Zhang C. (2017). Size effects of chitooligomers with certain degrees of polymerization on the chilling tolerance of wheat seedlings. Carbohydr. Polym..

[18] Gonzalez A., Castro J., Vera J., Moenne A. (2013). Seaweed oligosaccharides stimulate plant growth by enhancing carbon and nitrogen assimilation, basal metabolism, and cell division. J. Plant Growth Regul..

[19] Sarfaraz A., Naeem M., Nasir S., Idrees M., Aftab T., Hashmi N., Khan M.A.A., Varshney M., Varshney L. (2011). An evaluation of the effects of irradiated sodium alginate on the growth, physiological activities and essential oil production of fennel (*Foeniculum vulgare* Mill.). J. Med. Plants Res..

[20] Aftab T., Khan M.M.A., Idrees M., Naeem M., Hashmi N., Varshney L. (2011). Enhancing the growth, photosynthetic capacity and artemisinin content in *Artemisia annua* L. by irradiated sodium alginate. Radiat. Phys. Chem..

[21] Bi F., Iqbal S., Arman M., Ali A., Hassan M.-U. (2011). Carrageenan as an elicitor of induced secondary metabolites and its effects on various growth characters of chickpea and maize plants. J. Saudi Chem. Soc..

[22] Naeem M., Idrees M., Aftab T., Khan M.M.A., Moinuddin L., Varshney L. (2012). Depolymerised carrageenan enhances physiological activities and menthol production in *Mentha arvensis*. Carbohydr. Polym..

[23] Hashmi N., Khan M.M.A., Moinuddin Idrees M., Khan Z.H., Ali A., Varshney L. (2012). Depolymerized carrageenan ameliorates growth, physiological attributes, essential oil yield and active constituents of *Foeniculum vulgare* Mill. Carbohydr. Polym..

[24] Gonzalez A., Contreras R.A., Moenne A. (2013). Oligo-carrageenans enhance growth and contents of cellulose, essential oils and polyphenolic compounds in *Eucalyptus globulus* trees. Molecules.

[25] Cavallaro V., Patanè C., Cosentino S.L., Di Silvestro I., Copani V. (2014). Optimizing in vitro large scale production of giant reed (*Arundo donax* L.) by liquid medium culture. Biomass Bioenergy.

[26] Masondo N.A., Aremu A.O., Finnie J.F., Van Staden J. (2015). Growth and phytochemical levels in micropropagated *Eucomis autumnalis* subspecies *autumnalis* using different gelling agents, explant source, and plant growth regulators. In Vitro Cell. Dev. Biol. Plant.

[27] Jain R., Babbar S.B. (2006). Xanthan gum: An economical substitute for agar in plant tissue culture media. Plant Cell Rep..

[28] Bartkowiak A., Startek L., Salachna P., Zurawik P. (2008). Method of Hydrogel Coating Formation on the Surface of Plant Organs. Patent.

[29] Bartkowiak A., Hunkeler D. (1999). New microcapsules based on oligoelectrolyte complexation. Ann. N. Y. Acad. Sci..

[30] Soból M., Bartkowiak A., de Haan B., de Vos P. (2013). Cytotoxicity study of novel water-soluble chitosan derivatives applied as membrane material of alginate microcapsules. J. Biomed. Mater. Res. A.

[31] Startek L., Bartkowiak A., Salachna P., Kaminska M., Mazurkiewicz-Zapalowicz K. (2005). The influence of new methods of corm coating on freesia growth, development and health. Acta Hortic..

[32] Salachna P., Zawadzińska A., Wilas J. (2015). The use of natural polysaccharides in *Eucomis autumnalis* (Mill.) Chitt. propagation by twin-scale cuttings. Acta Hortic..

[33] Benschop M., Kamenetsky R., Le Nard M., Okubo H., De Hertogh A. (2010). The global flower bulb industry: Production, utilization, research. Hort. Rev..

[34] Salachna P., Zawadzińska A. (2013). The effects of flurprimidol concentrations and application methods on *Ornithogalum saundersiae* Bak. grown as a pot plant. Afr. J. Agric. Res..

[35] Salachna P., Zawadzińska A., Podsiadło C. (2016). Response of *Ornithogalum saundersiae* Bak. to salinity stress. Acta Sci. Pol.-Hortorum Cultus.

[36] Morzycki J., Wojtkielewicz A. (2005). Synthesis of a highly potent antitumor saponin OSW-1 and its analogues. Phytochem. Rev..

[37] Iguchi T., Kuroda M., Naito R., Watanabe T., Matsuo Y., Yokosuka A., Mimaki Y. (2017). Structural characterization of cholestane rhamnosides from *Ornithogalum saundersiae* bulbs and their cytotoxic activity against cultured tumor cells. Molecules.

[38] Salachna P., Wilas J., Zawadzińska A. (2015). The effect of chitosan coating of bulbs on the growth and flowering of *Ornithogalum saundersiae*. Acta Hortic..

[39] Barrera Necha L.L., Bautista-Baños S., Bautista-Baños S., Romanazzi G., Jiménez-Aparicio A. (2016). Prospects for the use of chitosan and other alternatives in ornamental conservation. Chitosan in the Preservation of Agricultural Commodities.

[40] Ma L., Li Y., Yu C., Wang Y., Li X., Chen Q., Bu N. (2012). Alleviation of exogenous oligochitosan on wheat seedlings growth under salt stress. Protoplasma.

[41] Bot J.L., Benard C., Robin C., Bourgaud F., Adamowicz S. (2009). The ‘trade-off’ between synthesis of primary and secondary compounds in young tomato leaves is altered by nitrate nutrition: Experimental evidence and model consistency. J. Exp. Bot..

[42] Cai Z., Kastell A., Mewis I., Knorr D., Smetanska I. (2012). Polysaccharide elicitors enhance anthocyanin and phenolic acid accumulation in cell suspension cultures of *Vitis vinifera*. Plant Cell Tissue Organ Cult..

[43] Hadwiger L.A. (2015). Anatomy of a nonhost disease resistance response of pea to *Fusarium solani*: PR gene elicitation via DNase, chitosan and chromatin alterations. Front. Plant Sci..

[44] Salachna P., Byczyńska A., Jeziorska I., Udycz E. (2017). Plant growth of *Verbena bonariensis* L. after chitosan, gellan gum or iota-carrageenan foliar applications. World Sci. News.

[45] Chevreau E., Mourgues F., Neveu M., Chevalier M. (1997). Effect of gelling agents and antibiotics on adventitious bud regeneration from in vitro leaves of pear. In Vitro Cell. Dev. Biol. Plant.

[46] Klimaszewska K., Smith D.R. (1997). Maturation of somatic embryos of *Pinus strobus* is promoted by a high concentration of gellan gum. Physiol. Plant..

[47] Tetsumura T., Tsukuda K., Kawase K. (2002). Micropropagation of Shinano walnut (*Juglans regia* L.). J. Jpn. Soc. Hortic. Sci..

[48] Scherer P.A., Müller E., Lippert H., Wolff G. (1988). Multielement analysis of agar and gelrite impurities investigated by inductively coupled plasma emission spectrometry as well as physical properties of tissue culture media prepared with agar or the gellan gum gelrite. Acta Hortic..

[49] Hadrami E., Housti F., Miehaux-Ferriere N., Carron M.P., D’Auzac J. (1993). Effects of gelling agents and liquid medium on embryogenie potential, polyamines and enzymatic factors in browning in *Hevea brasiliensis* calli. J. Plant Physiol..

[50] Lichtenthaler H.K., Wellburn A.R. (1983). Determinations of total carotenoids and chlorophylls a and b of leaf extracts in different solvents. Biochem. Soc. Trans..

[51] (1990). AOAC Official Methods of Analysis of the Association of Official Analytical Chemists.

[52] Singleton V.L., Rossi J.A. (1965). Colorimetry of total phenolics with phosphomolybdic-phosphotungstic acid reagents. Am. J. Enol. Vitic..

[53] Yen G.C., Chen H.Y. (1995). Antioxidant activity of various tea extracts in relation to their antimutagenicity. J. Agric. Food Chem..

[54] Rossi M., Giussani E., Morelli R., Scalzo R., Nani R.C., Torreggiani D. (2003). Effect of fruit blanching on phenolics and radical scavenging activity of highbush blueberry juice. Food Res. Int..

[55] Ostrowska A., Gawliński S., Szczubiałka Z. (1991). Methods for Analyzing and Assessing the Properties of Soil and Plants.

